# Tumors Following Trauma

**Published:** 1918-12

**Authors:** 


**Tumors Following Trauma.** By Dr. J. Vitrac. *Bulletin de la Société de Chirurgie de Paris*, May 7, 1918.

Can wounds be the cause of tumors in the injured tissue? Tumors have often appeared after trauma. It may be said that they would have developed even without the wound. It appears, however, that the cells of the organism may in certain cases be too active, and after having repaired the injured tissues, may continue to develop and so produce neoplasms.

Dr. Vitrac describes two cases in which tumors of the sarcoma variety appeared in intimate connection with a wound. A soldier had been wounded in the right thigh by a shell-fragment. This entered through the external side of the thigh, beneath the large trochanter, and penetrated into the popliteal space, without injuring any vessels or nerves. The projectile was removed, and the wound healed after much suppuration. One month after, a painless and hard tumor, about the size of a nut, was detected in the popliteal space. It increased slowly; after 5 months it was 15 cm. long and 8 cm. wide. Dr. Vitrac removed it then. Adherences had intruded between the tumor and the muscles, these appearing somewhat degenerated. The tumor proved to be an *angiosarcoma*.

The other case described by Dr. Vitrac resembles this one, the tumor appearing after severe bruises on the thigh.

